# MCOAN: multimodal contrastive representation learning for cross-omics adaptive disease regulatory network prediction

**DOI:** 10.1093/bioinformatics/btag033

**Published:** 2026-01-19

**Authors:** Junqi Long, Bo Liu, Jianqiang Li, Shuangtao Zhao

**Affiliations:** School of Computer Science, Beijing University of Technology, Beijing 100124, China; School of Mathematical and Computational Sciences, Massey University, Auckland 0745, New Zealand; School of Computer Science, Beijing University of Technology, Beijing 100124, China; Department of Thoracic Surgery, Beijing Tuberculosis and Thoracic Tumor Research Institute/Beijing Chest Hospital, Capital Medical University, Beijing 101149, China

## Abstract

**Motivation:**

Interactions among long noncoding RNAs, circular RNAs, microRNAs, and messenger RNAs form complex gene expression regulatory networks, which are of great significance for the diagnosis, prevention, and treatment of complex diseases. Although existing computational methods have been developed to predict interactions among certain molecular types, they are generally limited to single-modality perspectives, overlooking competitive specificity and co-target cooperativity across multi-omics molecules, and thereby limiting their ability to elucidate cross-omics regulatory mechanisms.

**Results:**

We proposed a novel cross-omics adaptive multimodal contrastive learning framework (MCOAN) that learns multimodal regulatory mechanisms and effectively predicts disease-associated molecular regulatory networks. Specifically, we first constructed a five-layer heterogeneous graph architecture to comprehensively integrate the complex regulatory associations among multi-omics nodes. Then, we proposed an unsupervised multimodal contrastive learning strategy that maximizes mutual information across distinct regulatory views, thereby enhancing node representations by efficiently capturing local neighborhood structure and global semantic information. Meanwhile, we also proposed a cross-omics adaptive learning mechanism that captures complex competitive specificity and co-target cooperativity across distinct regulatory networks, thereby further enhancing the structural awareness in node representations. Furthermore, we evaluated multiple downstream classifiers to accurately predict multimodal molecular regulatory networks. Finally, extensive experiments show that MCOAN consistently outperforms existing methods, achieving strong predictive accuracy and generalization (max AUC = 0.9881; max AUPR = 0.9826), and further confirm its real-world predictive performance through case studies.

**Availability and implementation:**

All resources are available at https://github.com/JunqiLab/MCOAN.git.

## 1 Introduction

Long noncoding RNAs (lncRNAs), circular RNAs (circRNAs), microRNAs (miRNAs), and messenger RNAs (mRNAs) exhibit complex multilayered mechanisms regulating gene expression and play an indispensable role in the initiation and progression of diseases (Tay et al. 2014, Yan et al. 2025). Substantial experimental evidence indicates that interactions among these molecules exert both synergistic and competitive influences on disease regulation (Schmitz et al. 2014, Tay et al. 2014, Denzler et al. 2016). However, existing biological experimental approaches exhibit limited predictive range and accuracy in elucidating multimolecular regulatory associations in disease, and their high costs preclude them from meeting the rapidly growing demand for predictions (Wang et al. 2022, Chai et al. 2025). It is, therefore, essential to develop efficient computational methods to complement multimolecular biological experiments.

With the rapid advancement in machine learning and deep learning in bioinformatics, numerous computational methods have been developed to predict intermolecular regulatory interactions. Early studies predominantly focused on machine learning approaches, which relied on handcrafted feature representations derived from molecular functional annotations, expression profiles, and sequences, and fed these features into supervised learning models for association prediction. For example, prior studies have also employed principal component analysis to project lncRNA and disease similarity features into a low-dimensional space, followed by random forest to predict molecular association scores (Zhu et al. 2021). These approaches offer high computational efficiency and a degree of interpretability, with tree-based models in particular revealing putative key factors via feature-importance measures. However, such methods rely heavily on representative features, struggle to automatically capture complex nonlinear regulatory patterns, and often overlook much of the topological information encoded within molecular networks, thereby compromising generalization and robustness.

Recently, many studies have adopted matrix factorization methods to decompose high-dimensional data matrices into the product of several low-rank matrices, thereby yielding more informative feature representations. For example, previous studies(Xiao et al. 2018, Li et al. 2021, Ding et al. 2022, Gao et al. 2022, Qin et al. 2024) employed decomposition techniques such as singular value decomposition (SVD), non-negative matrix factorization, and Laplacian Eigenmaps to compress high-dimensional and sparse molecular interactions into consistent latent-factor representations, effectively reducing feature dimensionality and alleviating data sparsity. However, such methods are essentially bilinear approximation methods that primarily rely on feature similarity and first-order topological structure, often focusing on single-relation prediction and struggling to capture nonlinear, multi-hop semantic regulatory information and higher-order topological structure, while their generalization performance is susceptible to data noise (Fu et al. 2018, Xiao et al. 2018, Wu et al. 2020). Meanwhile, other studies have also directly leveraged network topology for modeling regulatory interactions. Methods such as random walk with restart and label propagation (Nguyen et al. 2021, Qu et al. 2021, Chen et al. 2022, Yu et al. 2022, Chen et al. 2024) learn reachability probabilities among molecular nodes via multistep propagation on the normalized adjacency matrix, thereby enabling effective prediction of molecular regulatory links in sparse networks. Nevertheless, their heavy reliance on linear propagation mechanisms renders them sensitive to hyperparameters and prior graph structure, thereby constraining generalization across multimodal heterogeneous edges and hindering the effective capture of higher-order topology and high-dimensional heterogeneous interactions.

Moreover, graph neural networks have progressively incorporated attention mechanisms and message passing for heterogeneous graphs, with training generally relying on large numbers of labeled samples and substantial computational resources to supervise representation learning across multiple molecular types to improve structural awareness and semantic discriminability in deep learning. For example, recent studies (Sheng et al. 2022, Sheng et al. 2023, Chai et al. 2025) employed a graph contrastive learning strategy to learn unified molecular representations and designed a multichannel attention mechanism to capture molecular representations across inter-graph, intra-graph, and complex-graph aggregations. Further work (Wu et al. 2021, He et al. 2024) learned molecular node representations using graph auto-encoders and incorporated discriminators to determine their associative interactions. Nevertheless, these methods are prone to overfitting in weakly labeled multi-omics molecular regulatory networks. Their representation learning remains largely confined to local receptive fields and lacks effective alignment of global multimolecular regulatory semantics, thereby making it difficult to accurately capture the complex dynamic regulatory mechanisms across multimodal molecular regulatory networks.

To address the above limitations, the proposed MCOAN framework introduces innovations across three parts—structure, learning, and fusion. In the structural part, we comprehensively integrate interlayer interactions and intralayer multidimensional similarities among multi-omics molecules, constructing a five-layer heterogeneous graph comprising diseases, lncRNAs, circRNAs, miRNAs, and mRNAs, and uniformly mapping competitive specificity and co-target cooperativity across multi-omics molecules onto a learnable higher-order graph representation. In the learning part, we design multimodal unsupervised contrastive objectives to align representations between the local neighborhood structural view and the global semantic view, thereby effectively reducing dependence on strong supervision and improving the robustness of the learned representations. In the fusion part, we propose a cross-omics adaptive fusion strategy to achieve fine-grained dynamic modeling of multimodal molecular regulatory networks, yielding unified embeddings that are both discriminative and generalizable and providing a solid foundation for subsequent multi-omics prediction. In summary, the main contributions of MCOAN are as follows:

1. This study constructs a five-layer heterogeneous graph by comprehensively integrating inter-layer interactions and intra-layer multidimensional similarities among multi-omics molecules, enabling effective low-dimensional topological representations of multi-omics molecules.
2. MCOAN proposes a cross-omics adaptive learning mechanism to effectively learn competitive specificity and co-target cooperativity across multi-omics regulatory networks.
3. MCOAN proposes a multimodal contrastive representation learning strategy across multiple views to effectively capture global dependencies and local neighborhood structure in multi-omics molecular representations.
4. Extensive experiments demonstrate that the MCOAN framework outperforms existing methods, and case studies further validate its practical effectiveness for identifying multi-omics molecular regulatory networks.

## 2 Materials and methods

**Figure 1:**
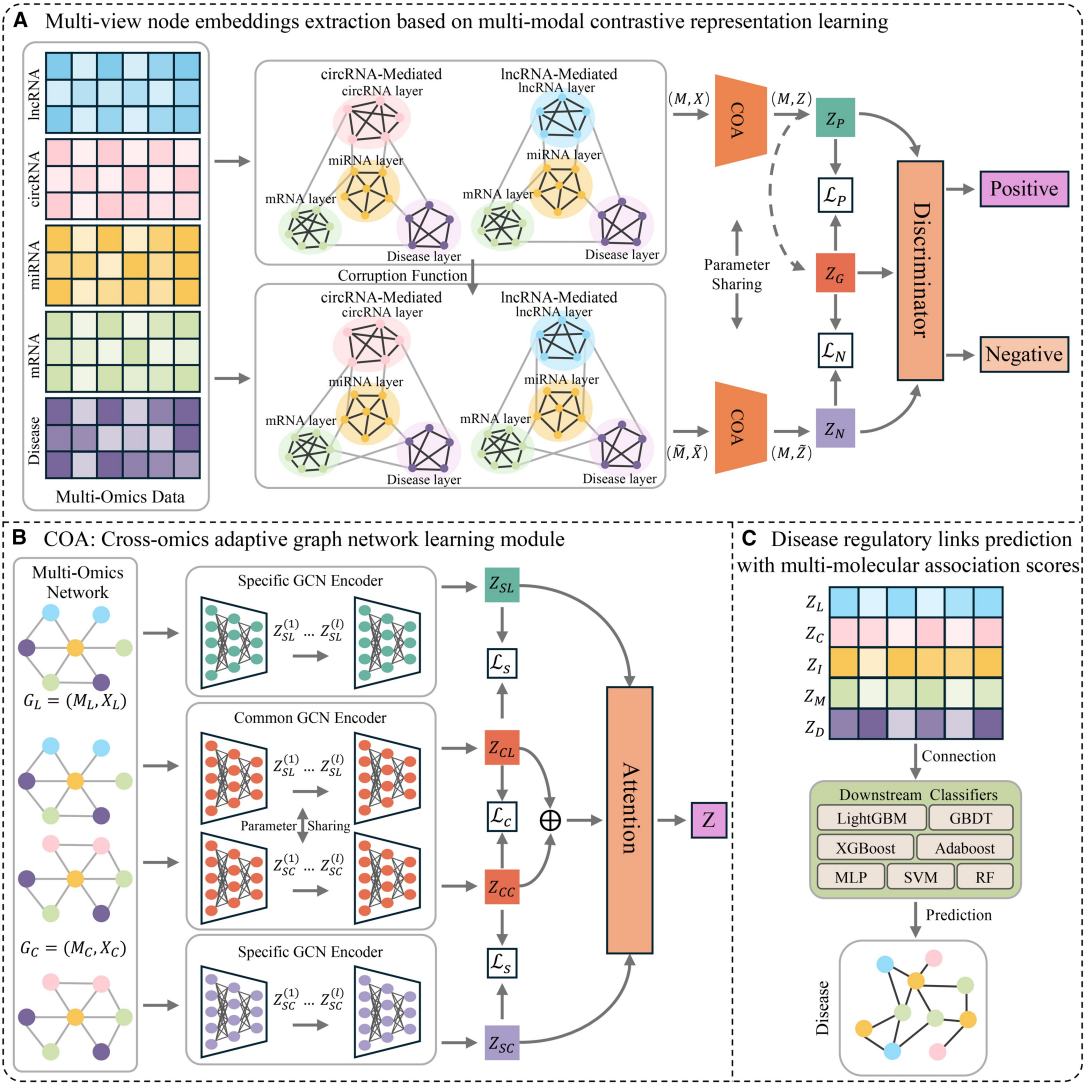
The overall framework of MCOAN. (A) Multi-view embedding feature extraction based on multimodal contrastive representation learning strategy. (B) Cross-omics adaptive regulatory learning mechanism. (C) Multi-molecule disease regulatory link prediction.

The overview of the proposed cross-omics adaptive disease regulatory network prediction framework based on multimodal contrastive learning was presented in Fig. 1. First, the framework comprehensively integrated multimodal inter-layer interactions and multidimensional intra-layer similarities among lncRNAs, circRNAs, miRNAs, mRNAs, and diseases to construct a five-layer heterogeneous disease regulatory network that encapsulates comprehensive information on intermolecular interactions (Fig. 1A). Next, the framework employed a multimodal self-supervised contrastive learning strategy with a cross-omics adaptive regulatory mechanism to effectively learn representative features of multi-omics molecular nodes (Fig. 1A and B). Finally, we constructed pairwise node embeddings and selected the optimal classifier to predict their molecular interaction probabilities within the multimolecular disease regulatory network (Fig. 1C).

### 2.1 Multi-modal molecular regulatory graph construction

To obtain more representative multi-omics regulatory interaction matrices, we constructed a five-layer multimodal regulatory heterogeneous graph (MORHG) based on inter-layer interactions and multidimensional intra-layer similarities among lncRNAs, circRNAs, miRNAs, mRNAs, and diseases. MORHG was defined as an undirected graph G=(V,E), where $V=\{V_{\mathrm{lncRNA}}\cup V_{\mathrm{circRNA}}\cup V_{\mathrm{miRNA}}\cup V_{\mathrm{mRNA}}\cup V_{\mathrm{disease}}\}$ is the set of all nodes, and $e_{ij}=(v_{i}{,v}_{j})\in E$ denotes an edge between nodes $v_{i}$ and $v_{j}$. We defined M=(A,S) as the composite adjacency matrix of G, where A is the multi-omics interactions matrix (1) and S is the multidimensional intra-layer similarity matrix (2).


(1) A={ALC∈RNl×Nc,(vi, vj)∈VlncRNA×VcircRNAALI∈RNl×Ni,(vi, vj)∈VlncRNA×VmiRNAALM∈RNl×Nm,(vi, vj)∈VlncRNA×VmRNAALD∈RNl×Nd, (vi, vj)∈VlncRNA×VdiseaseACI∈RNc×Ni,(vi, vj)∈VcircRNA×VmiRNAACM∈RNc×Nm,(vi, vj)∈VcircRNA×VmRNAACD∈RNc×Nd,(vi, vj)∈VcircRNA×VdiseaseAIM∈RNi×Nm,(vi, vj)∈VmiRNA×VmRNAAID∈RNi×Nd,(vi, vj)∈VmiRNA×VdiseaseAMD∈RNm×Nd,(vi, vj)∈VmRNA×Vdisease

In the inter-layer interactions matrix A, $N_{l},N_{c},N_{i},N_{m}$ and $N_{d}$ denote the numbers of lncRNAs, circRNAs, miRNAs, mRNAs, and diseases, respectively. For a node pair $\left(v_{i}{,v}_{j}\right)$, if an association $e_{ij}$ exists, the corresponding element was set to $A_{ij}=1$. Otherwise, $A_{ij}=0$.


(2) S={SL∈RNl×Nl, (vi,vj)∈VlncRNA×VlncRNA SC∈RNc×Nc,(vi,vj)∈VcircRNA×VcircRNASI∈RNi×Ni,(vi,vj)∈VmiRNA×VmiRNASM∈RNm×Nm,(vi,vj)∈VmRNA×VmRNASD∈RNd×Nd,(vi,vj)∈Vdisease×Vdisease

In the intra-layer multidimensional similarity matrix S, considering that RNA molecules generally exhibit functional homology and regulatory similarity patterns during disease progression, we defined a unified RNA intra-layer similarity representation $S^{K}(K\in \{L,\;C,\;I,\;M\})$ following Equation (3) (Sheng *et al.* 2023), which integrates the functional similarity F and the Gaussian interaction profile kernel (GIPK) similarity GK for lncRNAs, circRNAs, miRNAs, and mRNAs. Meanwhile, we integrated the disease semantic similarity SD and GIPK similarity ${GK}^{D}$  (4) to construct the intra-layer similarity of disease nodes. In these formulations, ${GK}_{R,ij}^{K}$ denote the GIPK similarity between nodes $v_{i}$ and $v_{j}$ of type K within modality R.


(3) SijK={FijK ,FijK ≠0GKL,ijK+GKC,ijK+GKI,ijK+GKM,ijK4,FijK=0

(4) $$S_{ij}^{D}=\frac{{SD}_{ij}+({GK}_{L,ij}^{D}+{GK}_{C,ij}^{D}+{GK}_{I,ij}^{D}+{GK}_{M,ij}^{D})/4}{2}$$

Accordingly, the MORHG adjacency matrix that comprehensively integrates inter-layer interactions and multidimensional intra-layer similarities was given by Equation (5), where $N_{v}=N_{l}+N_{c}+N_{i}+N_{m}+N_{d}$ and $A^{T}$ denotes the transpose of A. In addition, we defined the normalized form of M as the attribute matrix of MORHG, denoted $X\in R^{N_{v}\times N_{v}}$.


(5) $$M=[\begin{array}{ccccc} S^{L} & A^{LC} & A^{LI} & A^{LM} & A^{LD}\\ A^{{LC}^{T}} & S^{C} & A^{CI} & A^{CM} & A^{CD}\\ A^{{LI}^{T}} & A^{{CI}^{T}} & S^{I} & A^{IM} & A^{ID}\\ A^{{LM}^{T}} & A^{{CM}^{T}} & A^{{IM}^{T}} & S^{M} & A^{MD}\\ A^{{LD}^{T}} & A^{{CD}^{T}} & A^{{ID}^{T}} & A^{{MD}^{T}} & S^{D} \end{array}]\in R^{N_{v}\times N_{v}}$$

### 2.2 Cross-omics adaptive regulatory learning mechanism

First, to effectively align the two distinct biological regulatory networks, we construct two independent cross-omics regulatory subgraphs $G_{L}(M_{L},X_{L})$ and $G_{C}(M_{C},X_{C})\;$on the basis of the unified MORHG representation by masking molecular node relations that fall outside their respective regulatory scopes. Specifically, for the lncRNA-mediated (or circRNA-mediated) graph $G_{L}$(or $G_{C}$), all matrix contributions associated with circRNA (or lncRNA) nodes are masked, thereby ensuring that every retained relation in $M_{L}$(or $M_{C}$) and $X_{L}$(or $X_{C}$) belongs to the lncRNA (or circRNA) regulatory domain, with the non-zero interactions in $M_{L}$(or $M_{C}$) defined as the edge set of $G_{L}$(or $G_{C}$), ensuring that the two subgraphs form cross-omics aligned regulatory structures.

Then, to effectively learn the biological mechanisms by which multi-omics molecules regulate disease, we designed a cross-omics adaptive graph learning module to dynamically capture the multidimensional specific and cooperative molecular representations in multi-omics disease regulatory networks. The module comprises three parts: specificity encoder, common encoder, and attention mechanism (Fig. 1B). These two types of encoders are both defined on the lncRNA-mediated regulatory networks $G_{L}$ and the circRNA-mediated regulatory networks $G_{C}$, where the specificity encoder use independently parameterized graph convolutional networks to learn the distinct competitive regulatory patterns inherent to each network and produce the specificity embeddings $Z_{\mathrm{SL}}$ and $Z_{\mathrm{SC}}$; whereas the common encoder shares parameters across both networks to capture their cross-omics co-targeting regulatory pattern and produce the cooperative embeddings $Z_{\mathrm{CL}}$ and $Z_{\mathrm{CC}}$. The multi-dimensional attention mechanism (6) computes a weighted fusion of $Z_{\mathrm{SL}}$, $Z_{\mathrm{SC}}$, and $Z_{C\;}$ (the mean of $Z_{\mathrm{CL}}$ and $Z_{\mathrm{CC}}$) to obtain the final representation Z  (7), which are parameterized by attention weights $\alpha_{\mathrm{SL}}$,$\;\alpha_{\mathrm{SC}}\;$and $\alpha_{C}$, a learnable projection matrix W that maps embeddings into the attention space, a bias vector b, and a shared attention query vector q.


(6) $$\alpha_{s}=\frac{\exp\left(q^{T}\cdot \tanh\left(W\cdot Z_{s}+b\right)\right)}{\sum_{s^{'}\in s}\exp\left(q^{T}\cdot \tanh\left(W\cdot Z_{s^{'}}+b\right)\right)}$$

(7) $$Z=\alpha_{\mathrm{SL}}\cdot Z_{\mathrm{SL}}+\alpha_{\mathrm{SC}}\cdot Z_{\mathrm{SC}}+\alpha_{C}\cdot Z_{C}$$

Next, to further ensure that the module effectively learns both competitive specificity and co-target cooperativity in multi-omics molecular regulation networks, we introduced a specificity regularization term $L_{S}$ and a consistency regularization term$\;L_{C}$. The specificity constraint $L_{S}$ is based on the Hilbert–Schmidt Independence Criterion (HSIC), which measures the discrepancy between the lncRNA-specific and circRNA-specific embedding spaces. For embeddings $Z_{C}$ and $Z_{S}$, HSIC is defined as:


(8) $$\mathrm{HSIC}(Z_{C},Z_{S})={\left(n-1\right)}^{-2}\cdot tr(RK_{C}RK_{S})$$

where $K_{C}$ and $K_{S}$ are Gram matrices with entries $K(i,j)=Z_{i}Z_{j}$, and $R=I-1/nee^{⊤}$is the centering matrix with identity matrix I and e is the all-one column vector. Based on this formulation, the specificity regularization is computed as:


(9) $$L_{s}=\mathrm{HSIC}(Z_{\mathrm{CL}},Z_{\mathrm{SL}})+\mathrm{HSIC}(Z_{\mathrm{CC}},Z_{\mathrm{SC}})$$

Finally, although the common encoder shares a unified parameter matrix, we introduce a consistency constraint $L_{C}$ to align its cross-omics cooperative embeddings by comparing the L2-normalized embeddings from the two common-encoder branches:


(10) $$L_{c}={∥Z_{\mathrm{CL}}\cdot Z_{\mathrm{CL}}^{T}-Z_{\mathrm{CC}}\cdot Z_{\mathrm{CC}}^{T}∥}_{2}^{2}$$

### 2.3 Multi-modal contrastive representation learning strategy

**Figure 2:**
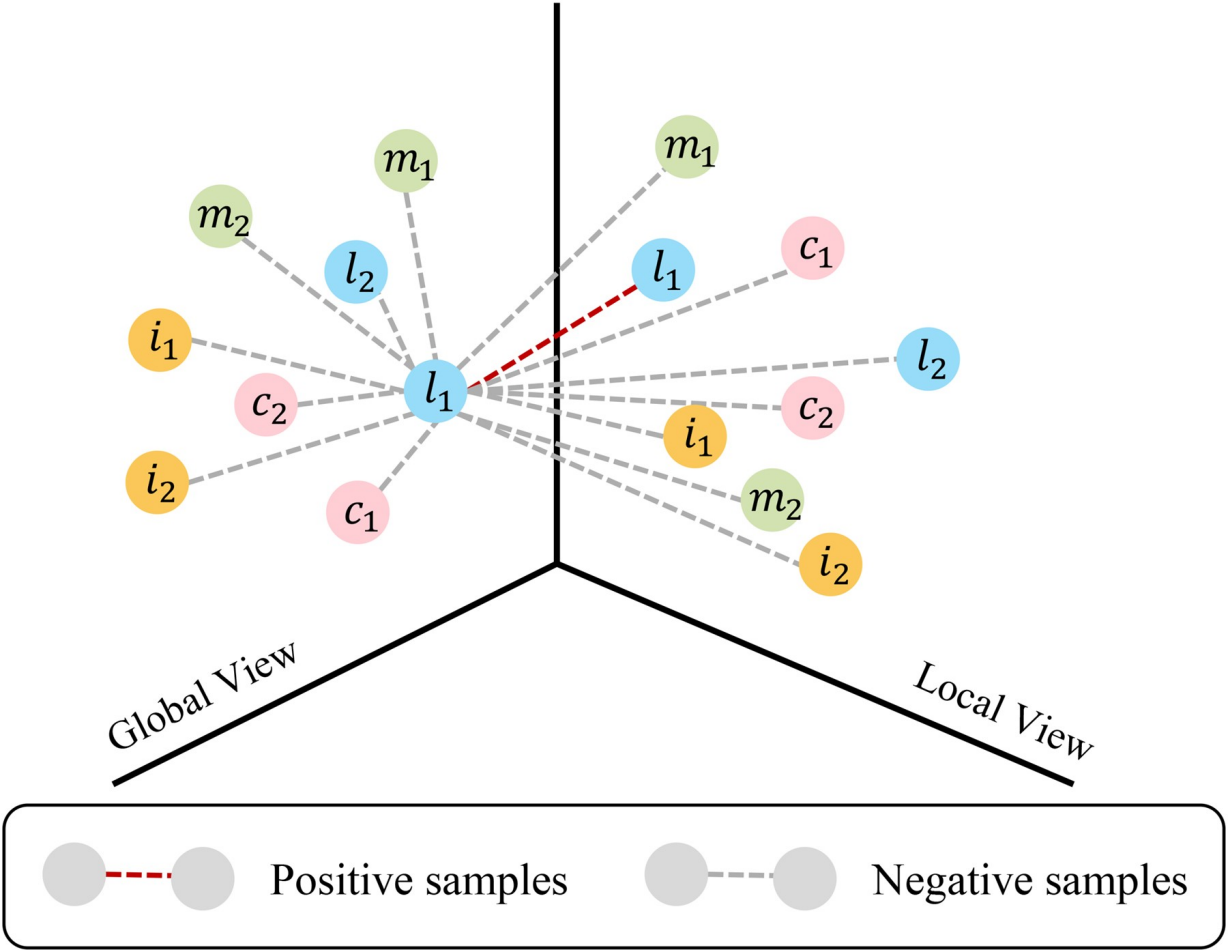
Multi-modal contrastive representation learning.

To comprehensively learn local neighborhood structural features and global semantic information in multimodal molecular regulatory networks, we adopted an unsupervised graph contrastive learning approach (Teng *et al.* 2025) together with binary cross-entropy (BCE) losses $L_{P}$ and $L_{N}$ (11 and 12) to capture salient multimodal molecular features (Fig. 1A). The learning objective employed a linear discriminator to maximize mutual information across distinct regulatory views (Fig. 2), thereby extracting more discriminative high-level node representations (13). In this formulation, both $Z_{N}$ and $Z_{P}$ are computed by the COA module directly from the unified MORHG representation, where $Z_{N}$ denotes the negative sample features extracted from the corrupted multimodal data $\tilde{(M},\tilde{X)}$ generated by a random corruption function, $Z_{P}$ denotes the local view features of positive samples, α and β are the regularization coefficients of the biologically positive COA mechanism, and $Z_{G}$ denotes the global semantic view computed following DGI (Veličković *et al.* 2019) by aggregating the $Z_{P}$ representations into a graph-level global embedding (Fig. 1A).


(11) $$L_{P}=-\frac{1}{N}\sum_{i=1}^{N}E_{\left(M,X\right)}[\log D(Z_{P,i},Z_{G})]$$

(12) $$L_{N}=-\frac{1}{N^{'}}\sum_{i=j}^{N^{'}}E_{\left(\tilde{M},\tilde{X}\right)}[\log(1-D(Z_{N,j},Z_{G}))]$$

(13) $$L=\frac{N}{N+N^{'}}L_{P}+\frac{N^{'}}{N+N^{'}}L_{N}+{\alpha L}_{S}+{\beta L}_{C}$$

## 3 Results

### 3.1 Comparison with other baseline methods

**Figure 3:**
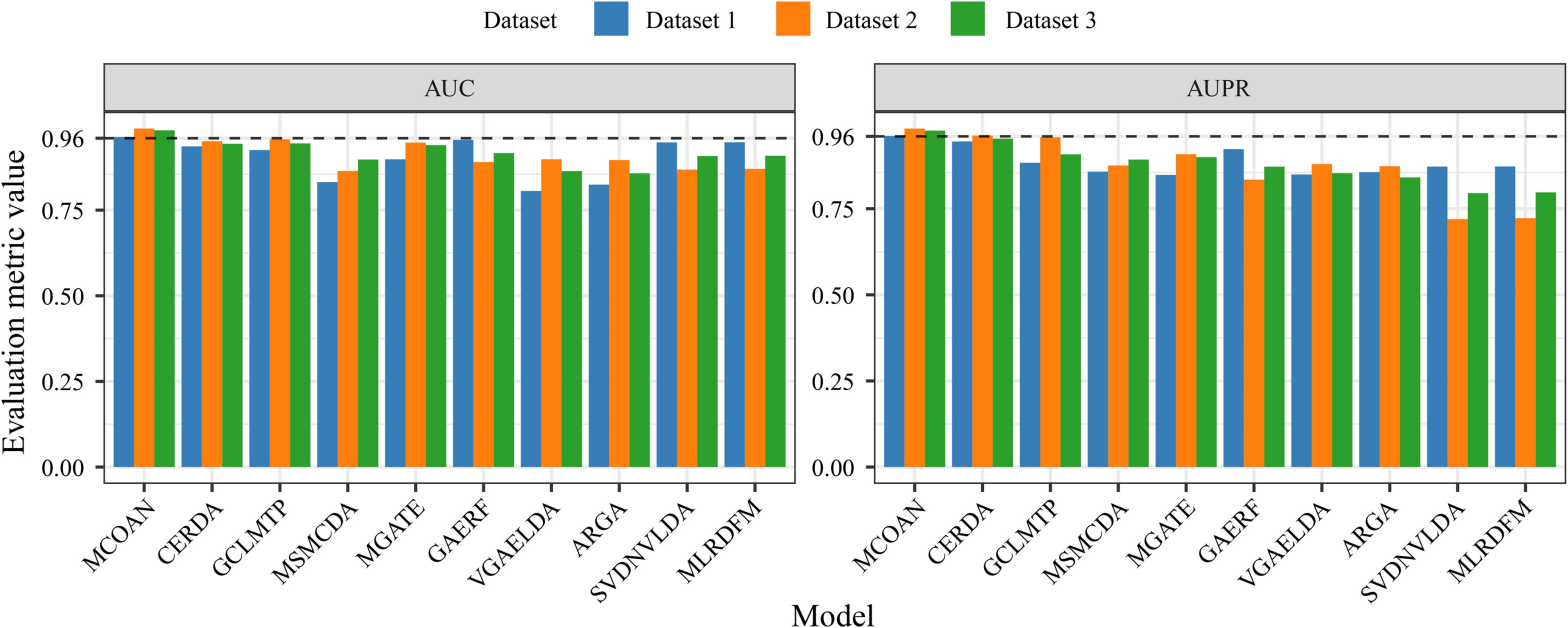
Performance comparison with other baseline methods.

To systematically assess the advantages of the proposed model for multi-omics regulatory network prediction, we conducted multiple performance comparisons between MCOAN and all baseline models across three independent datasets (Fig. 3). The results show that MCOAN consistently achieves the best performance across all datasets, exhibiting significant superiority over existing methods in both discriminative ability (AUC) and robustness to multimodal data imbalance (AUPR). Specifically, MCOAN achieves strong predictive performance (AUC = 0.9635 and AUPR = 0.9610) on Dataset 1. Similarly, MCOAN achieves the highest predictive performance (AUC = 0.9881 and AUPR = 0.9826) on the largest dataset (Dataset 2), not only outperforming typical matrix factorization methods (e.g. SVDNVLDA) but also significantly outperforming deep learning models (e.g. CERDA). Moreover, MCOAN maintains robust predictive performance (AUC = 0.9830, AUPR = 0.9767) on Dataset 3, achieving ∼2%–4% performance gains over the second-best model, exhibiting strong discriminative and generalization capabilities. Finally, to verify the model’s capability of learning from highly imbalanced multimodal data, we further evaluated performance on the three least frequent edge types in the more skewed Datasets 2 and 3. The results show that the AUC and AUPR remain at 0.9796 and 0.9805 in Dataset 2, respectively, while the AUC and AUPR reach 0.9351 and 0.9332 in Dataset 3, consistently exhibiting stable and significant advantages over all baseline methods.

### 3.2 Ablation study

#### 3.2.1 The impact of multi-dimensional similarity matrices

**Figure 4:**
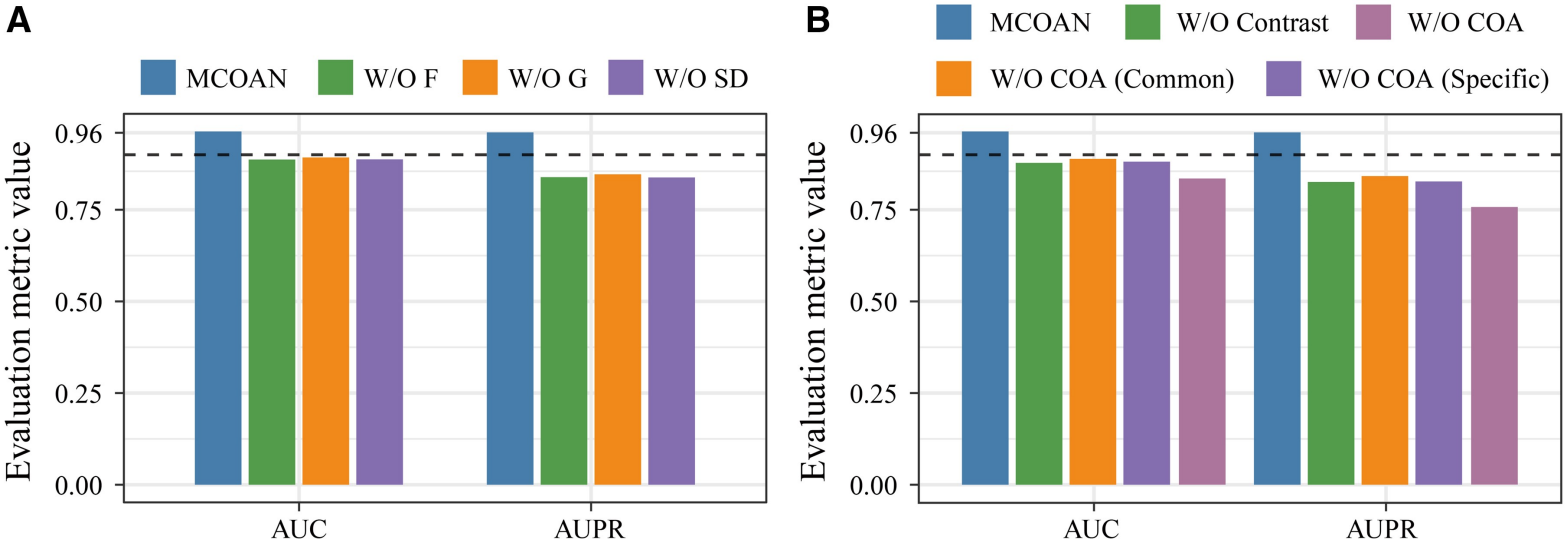
Multi-perspective ablation validation. (A) Ablation validation of the multi-dimensional similarity matrices. (B) Ablation validation of the feature learning strategies.

To investigate the impact of different similarity matrices, we performed fivefold cross-validation to compare ablated variants that remove each similarity source. The results show that MCOAN achieves the best performance on both AUC and AUPR, significantly outperforming all variants (Fig. 4A). Specifically, removing the multi-omics GIPK similarity (W/O G) or the disease semantic similarity (W/O SD) from MCOAN leads to marked drops in both performance metrics. Moreover, removing the functional similarity (W/O F) from MCOAN results in the largest degradation, with the most pronounced decline observed for AUPR. Therefore, these results demonstrate that the multi-dimensional similarity matrices are indispensable for link prediction, with functional similarity contributing most to predictive performance.

#### 3.2.2 The impact of feature learning strategies

To assess the effectiveness of the feature learning strategies, we conducted fivefold cross-validation to compare the ablated variants that each key strategy. The results show that MCOAN significantly outperforms all ablated variants (Fig. 4B). Specifically, removing the multimodal contrastive learning strategy leads to marked performance degradation, with AUC and AUPR decreasing by 8.56% and 13.52%, respectively. Similarly, ablating different regulatory mechanisms within the COA module also causes noticeable performance declines, especially when the specificity mechanism is removed. Furthermore, removing the COA module yields the greatest performance reduction, reducing AUC and AUPR by 12.83% and 20.39%, respectively. Overall, these results effectively highlight the critical contributions of different feature learning strategies to the representation learning on multimodal molecular regulatory data.

### 3.3 Data sensitivity analysis

**Figure 5:**
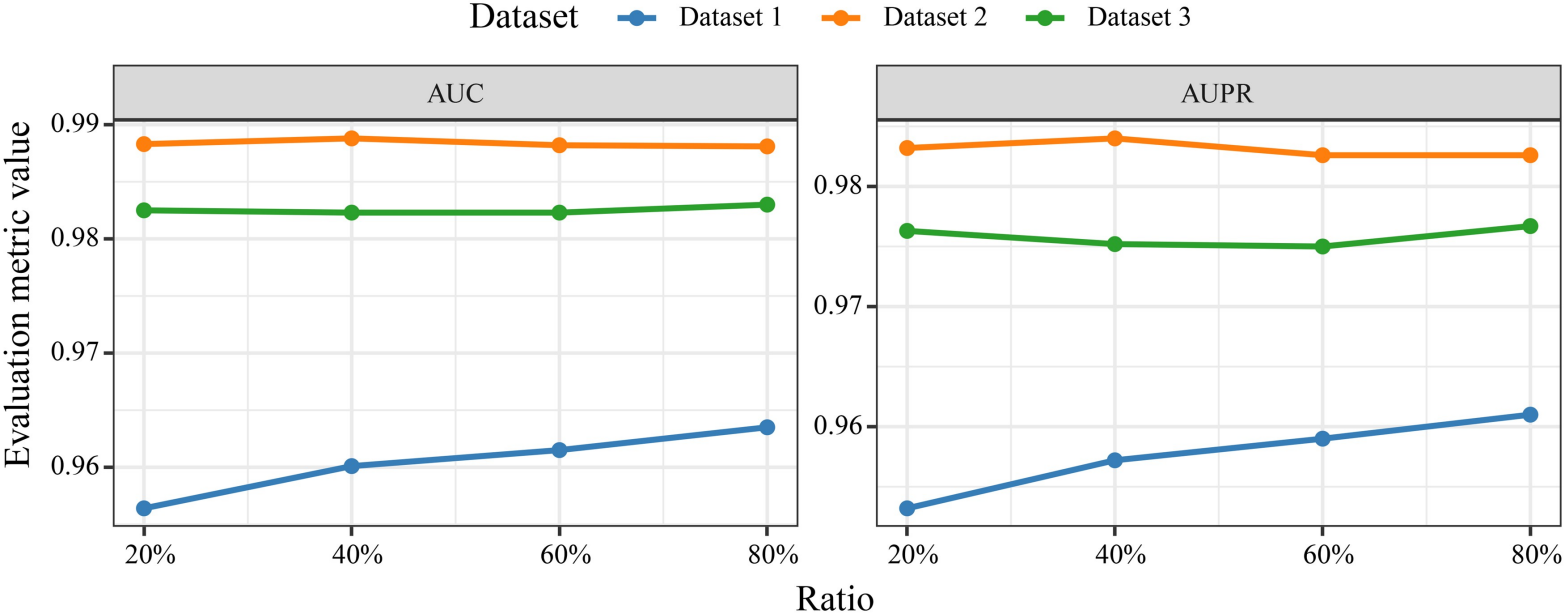
Data sensitivity analysis.

To evaluate the robustness of MCOAN under varying training data sizes, we conducted a sensitivity analysis by gradually reducing the proportion of each training set from 80% to 20%. The results show that the model’s predictive performance remains high overall across all three independent datasets, exhibiting only minor fluctuations (Fig. 5). Specifically, AUC decreases slightly from 0.9635 to 0.9564 and AUPR from 0.9610 to 0.9532 on Dataset 1. Moreover, AUC and AUPR remain ∼0.9881 and 0.9826 on Dataset 2. Similarly, AUC and AUPR also remain around 0.9830 and 0.9767 on Dataset 3. These findings indicate that MCOAN is minimally sensitive to training data size, maintaining strong predictive performance even with only 20% of the training samples and thereby effectively mitigating the impact of data limitations in practical applications.

### 3.4 Time complexity analysis

To systematically evaluate the computational scalability of MCOAN, we conducted a time complexity analysis. MCOAN comprises three core parts: the COA mechanism for cross-omics adaptive feature learning, contrastive learning for multimodal representation extraction, and a downstream classifier for molecular regulatory interaction prediction (Fig. 1). The COA module has overall complexity O(|E|·V), primarily driven by the specific GCN encoder and the shared GCN encoder. The multimodal contrastive learning and prediction stages have complexities $O(V^{2})$ and O(|E|·log⁡|E|), respectively. In summary, given the sparsity of real biological regulatory networks, the overall complexity can be expressed as $O(V^{2})$.

**Figure 6:**
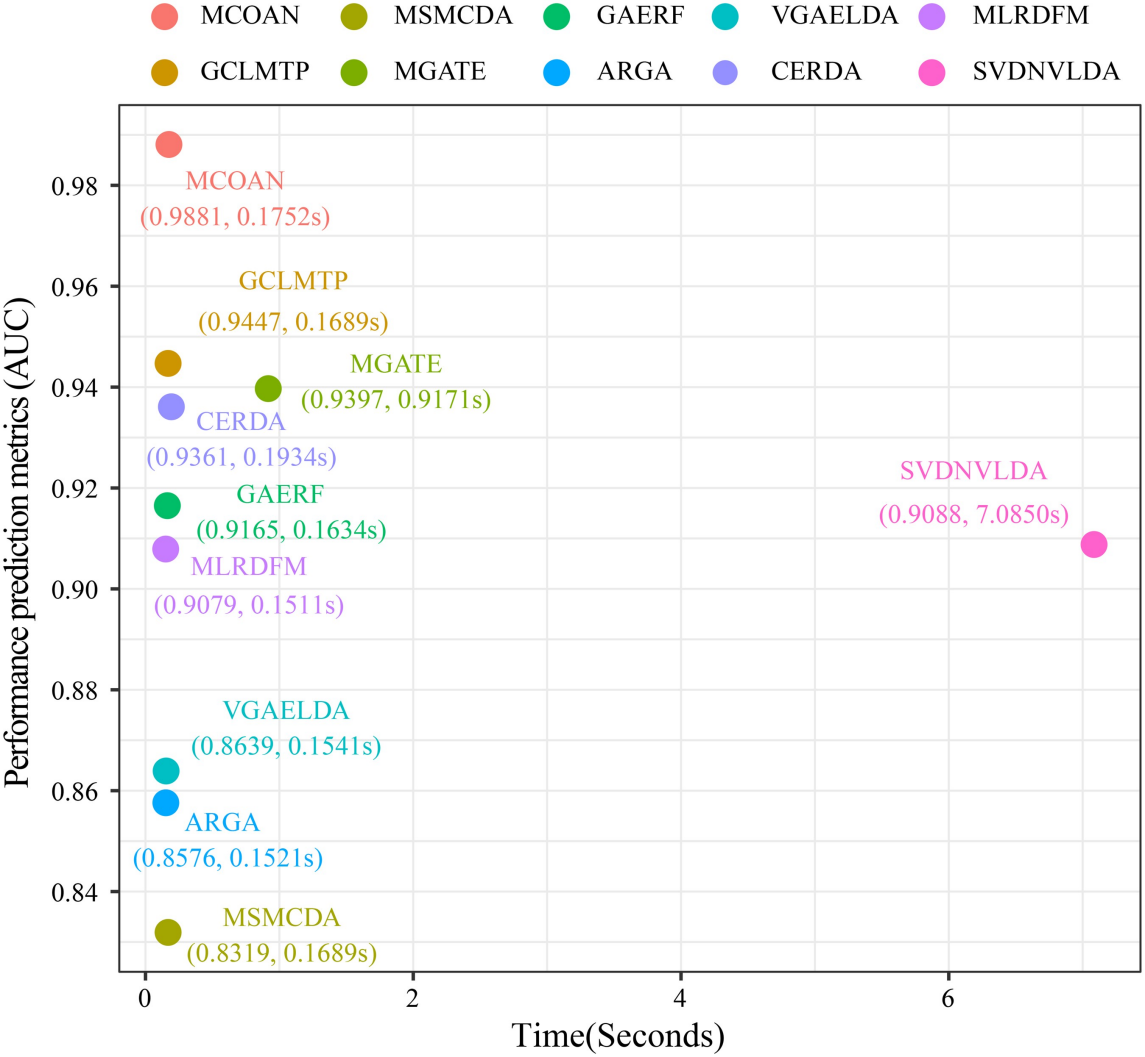
Computational complexity analysis.

To present the analysis results more intuitively, we further compared the training time and predictive performance of each baseline method on the largest dataset (Fig. 6). The results show that MCOAN achieves the highest predictive performance (AUC = 0.9881) while maintaining a low training cost (0.1752 s). In contrast, the traditional SVD-based methods require significantly more time for each matrix decomposition step. Moreover, although other baseline methods are close to MCOAN in efficiency, but they still exhibit significant gaps in predictive performance. Taken together, these results indicate that MCOAN strikes an effective balance between computational cost and predictive performance, demonstrating strong scalability and practical value.

### 3.5 Case study

**Figure 7:**
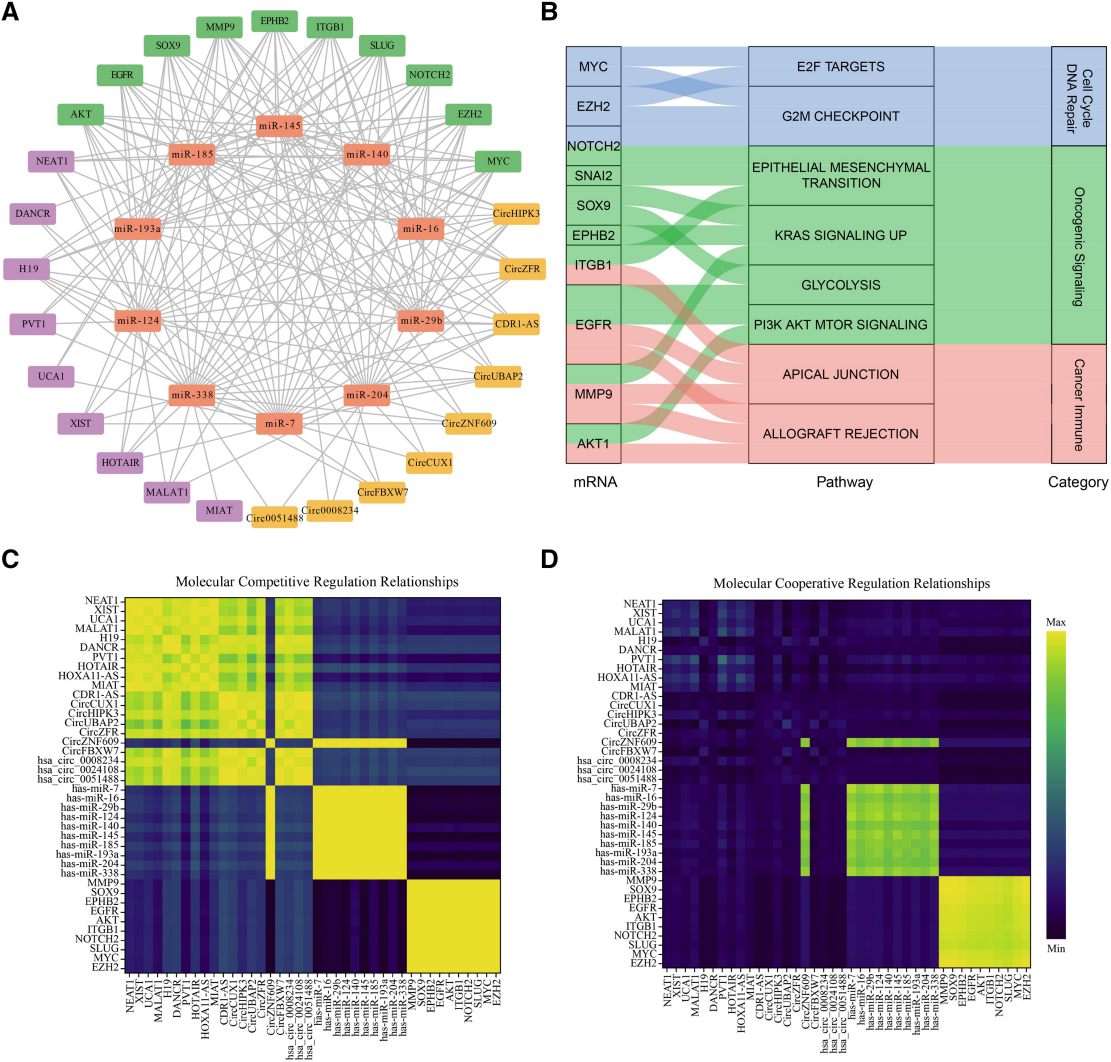
Multi-omics regulatory network prediction results. (A) Construction of a multi-omics regulatory network. (B) Functional enrichment analysis. (C) Molecular competitive regulation relationships. (D) Molecular cooperative regulation relationships.

To further validate the practicality of MCOAN, we conducted a case study on lung cancer, which has high global incidence and mortality (Siegel *et al.* 2025), to further explore potential multi-omics regulatory mechanisms. Specifically, we first used MCOAN to predict the top 10 cancer-related candidate genes within the multi-omics regulatory network (Table 5, available as supplementary data at *Bioinformatics* online). The results show that all candidates are supported by authoritative databases, indicating that the model reliably identifies regulatory genes from cross-omics data. We then constructed an interaction network (Fig. 7A) and performed biological functional enrichment analysis (Fig. 7B) on the predicted complex multi-omics regulatory network. The enriched pathways are mainly related to cell cycle and DNA repair (MYC, EZH2, NOTCH2), cancer signaling (NOTCH2, SNAI2, SOX9, EPHB2, ITGB1, EGFR, MMP9, AKT1), and cancer immunity (TGFB1, EGFR, MMP9, AKT1), and are consistent with prior reports (Liu et al. 2024, Song et al. 2024, Rocca et al. 2025).

Additionally, the network can robustly capture competitive specificity and co-target cooperativity, as evidenced by the visualized correlations and dependencies among top-ranked molecular features across multi-omics (Fig. 7C and D). The results showed significant correlations within each molecular layer and between the two mediator layers (lncRNA and circRNA) in the competitive regulatory heatmap, indicating that the model can effectively capture competitive relationships both across upstream regulatory networks and within individual molecular layers. In contrast, only miRNA and mRNA layers exhibited significant dependence in cooperative regulatory relationships, indicating that the model successfully identifies cooperative relationships in downstream molecular layers. The regulatory patterns inferred by MCOAN closely align with known ceRNA mechanisms. For example, miR-145 and miR-29b competitively regulate downstream targets via ceRNA interactions, whereas EGFR and ITGB1 exhibit coordinated expression within the PI3K/AKT/mTOR pathway (Haake et al. 2022, Gu et al. 2025, Liang et al. 2025, Wang et al. 2025a, 2025b). Overall, these findings demonstrate that MCOAN can effectively elucidate the complex multimolecular regulatory mechanisms of disease and facilitate the identification of potential therapeutic targets.

## 4 Discussion

This study proposed a cross-omics adaptive framework for disease regulatory network prediction based on multimodal contrastive representation learning (MCOAN). The framework combined cross-omics adaptive learning and multimodal contrastive representation learning strategies to dynamically learn competitive specificity and co-target cooperativity across multimodal regulatory networks, enabling systematic modeling of complex multi-omics disease mechanisms. Extensive experiments showed that MCOAN outperformed multiple baselines while remaining computationally efficient. Furthermore, the sensitivity analysis indicated MCOAN’s low dependence on training set size and robustness to data imbalance, reflecting strong discriminative power and generalization. In addition, MCOAN not only identified candidate genes with potential biological significance but also revealed signaling pathways and molecular interaction patterns closely associated with tumorigenesis and progression in the case study, thereby further validating the model’s clinical reliability and translational potential.

In summary, MCOAN demonstrated excellent predictive performance and scalability for cross-omics regulatory prediction tasks. It not only maintained robust modeling and prediction advantages across diverse imbalanced data distributions but also provided new avenues to interrogate complex multimodal molecular regulatory mechanisms and discover potential therapeutic targets. Future research will further integrate complex pathological structures, gene expression profiles, and other multidimensional clinical modalities to better evaluate and optimize the model, enhancing its applicability in real-world medical settings.

## Supplementary Material

btag033_Supplementary_Data

## Data Availability

The data used in this study were obtained from multiple publicly available databases, including LncACTdb v3.0 (http://bio-bigdata.hrbmu.edu.cn/LncACTdb), RNADisease v4.0 (http://www.rnadisease.org), NPInter v5.0 (http://bigdata.ibp.ac.cn/npinter5), LncRNA2Target v2.0 (https://bio-computing.hrbmu.edu.cn/lncrna2target), Circ2Disease (http://bioinformatics.zju.edu.cn/Circ2Disease), miRTarBase v10.0 (https://mirtarbase.cuhk.edu.cn), and CircBank (https://www.circbank.cn).
