# Supplementary material for: MCOAN: multimodal contrastive representation learning for cross-omics adaptive disease regulatory network prediction
Source: Bioinformatics. 2026 Jan 19;42(2):btag033. doi: 10.1093/bioinformatics/btag033 (PMC12881826; doi:10.1093/bioinformatics/btag033)
Supplement: btag033_Supplementary_Data [file btag033_supplementary_data.pdf]

Supplementary Information

SI 1. Extended Methods

SI 1. 1. Multi-molecular regulatory network prediction

To achieve accurate prediction of multimolecular regulatory networks, we uniformly modeled all candidate multi-omics node pairs $(u, v) \in F$ based on multimodal node embedding representation (Figure 1C). To identify the optimal downstream predictor, we systematically evaluated seven representative classifiers, including adaptive boosting (AdaBoost), extreme gradient boosting (XGBoost), gradient boosting decision tree (GBDT), lightweight gradient boosting machine (LightGBM), multilayer perceptron (MLP), random forest (RF) and support vector machine (SVM), and compared their predictive performance across datasets of varying sizes to comprehensively assess the applicability and robustness of the learned representations. In addition, to assess the biological interpretability of the predictions, we performed Gene Set Enrichment Analysis (GSEA) (Subramanian et al. 2005) on high-confidence links generated by the trained models, thereby further elucidating potential multi-omics regulatory mechanisms.

SI 2. Extended Results

SI 2.1. Construction of the experiment

Datasets

In this study, we systematically curated public resources and constructed three independent datasets to comprehensively evaluate the predictive performance of the proposed MCOAN framework, with provided summary statistics in Table S1. Specifically, Dataset 1 was derived from LncACTdb v3.0(Wang et al. 2022), and comprised 3,006 nodes and 15,217 interaction edges. Dataset 2 was constructed from RNADisease v4.0(Chen et al. 2023) and NPInter v5.0(Zheng et al. 2023), and comprised 4,739 nodes and 224,412 interaction edges as the largest dataset. Dataset 3 was constructed from multiple sources, including LncRNA2Target v2.0(Cheng et al. 2019), Circ2Disease(Yao et al. 2018), miRTarBase v10.0(Cui et al. 2025), and CircBank(Liu et al. 2019), and comprised 3,164 nodes and 111,867 interaction edges.

Experimental setup

The MCOAN framework was implemented in the PyTorch(Paszke et al. 2019) and trained on a workstation with an RTX 4090 GPU and 128 GB of memory. The learning rate (lr) was set to 0.01, α to 0.01, β to 0.1, the dropout rate to 0.3, and Adam was used as the optimizer. Parameter sensitivity analysis indicated that the model achieved superior performance when the COA base module adopted a two-layer GCN architecture with an embedding dimension of 64 (Tables S2–S3). In addition, to evaluate discriminative ability and robustness under class imbalance(Saito and Rehmsmeier 2015), we employed two metrics widely used for link prediction: the area under the receiver operating characteristic curve (AUC) and the area under the precision–recall curve (AUPR).

Baseline methods

To assess the framework, we compared MCOAN with nine existing prediction methods, including CERDA(Chai et al. 2025), MSMCDA(Zhang et al. 2025), GCLMTP(Sheng et al. 2023), MGATE(Sheng et al. 2022), GAERF(Wu et al. 2021), VGAELDA(Shi et al. 2021), ARGA(Pan et al. 2018), SVDNVLDA(Li et al. 2021), and MLRDFM(Ding et al. 2022). Because no models are tailored to multi-omics molecular network structures, we reran the publicly released code for these baselines on the multi-omics datasets to obtain the multimolecular regulatory network predictions.

SI 2.2. Ablation study

The impact of downstream classifiers

To evaluate the influence of downstream classifiers on predictive performance, we compared seven existing classifiers: RF, XGBoost, LightGBM, MLP, SVM, AdaBoost, and GBDT (Table S4). The results show that RF outperforms all alternatives across both evaluation metrics, improving over the second-best classifier by 0.46%–0.65% in AUC and 0.37%–0.79% in AUPR, thereby demonstrating the strong nonlinear modeling capacity and generalization of RF for the extracted multimodal regulatory representations.

SI 3. Supplementary Tables

Table S1. The statistics for each dataset

Type	Category	Dataset 1	Dataset 2	Dataset 3
Nodes	LncRNAs	892	383	386
	circRNAs	326	68	233
	miRNAs	659	869	1776
	mRNAs	1607	3216	651
	Diseases	122	203	118
Edges	Lis	2581	6728	1003
	LMs	2891	1771	831
	LDs	1477	2525	794
	Cis	357	122	17178
	CMs	413	11	233
	CDs	219	66	159
	Ims	3473	178891	90991
	IDs	1853	30502	95
	MDs	1953	3796	583

Table S2. Parametric sensitivity analysis of GCN layers

Number	AUC		AUPR	
	Train	Test	Train	Test
1	0.9677	0.9634	0.9651	0.9606
2	**0.9678**	**0.9635**	**0.9657**	**0.9610**
3	0.9651	0.9605	0.9633	0.9569
4	0.9681	0.9618	0.9655	0.9593

Table S3. Parametric sensitivity analysis of embedding sizes

Size	AUC		AUPR	
	Train	Test	Train	Test
32	0.9669	0.9599	0.9645	0.9568
64	**0.9678**	**0.9635**	**0.9657**	**0.9610**
128	0.9670	0.9632	0.9650	0.9607
256	0.9675	0.9635	0.9656	0.9609
512	0.9667	0.9620	0.9621	0.9596

Table S4. Performance comparison between different classifiers

Classifiers	Dataset 1		Dataset 2		Dataset 3	
	AUC	AUPR	AUC	AUPR	AUC	AUPR
RF	**0.9635**	**0.9610**	**0.9881**	**0.9826**	**0.9830**	**0.9767**
XGBoost	0.9557	0.9517	0.9830	0.9753	0.9720	0.9516
LightGBM	0.9589	0.9573	0.9812	0.9753	0.9765	0.9688
MLP	0.9552	0.9522	0.9805	0.9745	0.9749	0.9668
SVM	0.9338	0.9248	0.9714	0.9614	0.9498	0.9204
Adaboost	0.9342	0.9298	0.9729	0.9628	0.9574	0.9402
GBDT	0.9535	0.9511	0.9799	0.9734	0.9751	0.9671

Table S5. Prediction results of the top 10 candidate regulatory genes in lung cancer

Category	Node	Evidencen
lncRNA	NEAT1	LncACTdb v3.0,RNADisease v4.0,LncRNA2Traget v2.0
	XIST	LncACTdb v3.0,RNADisease v4.0,LncRNA2Traget v2.0
	UCA1	LncACTdb v3.0,RNADisease v4.0,LncRNA2Traget v2.0
	MALAT1	LncACTdb v3.0,RNADisease v4.0,LncRNA2Traget v2.0
	H19	LncACTdb v3.0,RNADisease v4.0,LncRNA2Traget v2.0
	DANCR	LncACTdb v3.0,RNADisease v4.0,LncRNA2Traget v2.0
	PVT1	LncACTdb v3.0,RNADisease v4.0,LncRNA2Traget v2.0
	HOTAIR	LncACTdb v3.0,RNADisease v4.0,LncRNA2Traget v2.0
	HOXA11-AS	LncACTdb v3.0,RNADisease v4.0,LncRNA2Traget v2.0
	MIAT	LncACTdb v3.0,RNADisease v4.0,LncRNA2Traget v2.0
circRNA	CDR1-AS	RNADisease v4.0
	CircCUX1	RNADisease v4.0
	CircHIPK3	RNADisease v4.0
	CircUBAP2	RNADisease v4.0
	CircZFR	RNADisease v4.0
	CircZNF609	RNADisease v4.0
	CircFBXW7	RNADisease v4.0; LncACTdb v3.0
	hsa_circ_0008234	LncACTdb v3.0
	hsa_circ_0024108	LncACTdb v3.0
	hsa_circ_0051488	LncACTdb v3.0
miRNA	has-miR-7	LncACTdb v3.0; LncRNA2Traget v2.0
	has-miR-16	LncACTdb v3.0; LncRNA2Traget v2.0
	has-miR-29b	LncACTdb v3.0; RNADisease v4.0
	has-miR-124	LncACTdb v3.0; LncRNA2Traget v2.0
	has-miR-140	LncACTdb v3.0; LncRNA2Traget v2.0
	has-miR-145	LncACTdb v3.0; RNADisease v4.0
	has-miR-185	LncACTdb v3.0
	has-miR-193a	LncACTdb v3.0; LncRNA2Traget v2.0
	has-miR-204	LncACTdb v3.0
	has-miR-338	LncACTdb v3.0; LncRNA2Traget v2.0
mRNA	MMP9	LncACTdb v3.0; LncRNA2Traget v2.0
	SOX9	LncACTdb v3.0
	EPHB2	LncACTdb v3.0
	EGFR	LncACTdb v3.0; RNADisease v4.0; LncRNA2Traget v2.0
	AKT	LncACTdb v3.0; LncRNA2Traget v2.0
	ITGB1	LncACTdb v3.0
	NOTCH2	LncACTdb v3.0
	SLUG	LncACTdb v3.0; LncRNA2Traget v2.0
	MYC	LncACTdb v3.0; LncRNA2Traget v2.0
	EZH2	LncACTdb v3.0; LncRNA2Traget v2.0

References

Chai, Z.*, et al.* (2025) Predicting disease associations based on the higher order structure of ceRNA networks. *Briefings in Bioinformatics*;26(5).

Chen, J.*, et al.* (2023) RNADisease v4.0: an updated resource of RNA-associated diseases, providing RNA-disease analysis, enrichment and prediction. *Nucleic Acids Res*;51(D1):D1397–d1404.

Cheng, L.*, et al.* (2019) LncRNA2Target v2.0: a comprehensive database for target genes of lncRNAs in human and mouse. *Nucleic Acids Res*;47(D1):D140–d144.

Cui, S.*, et al.* (2025) miRTarBase 2025: updates to the collection of experimentally validated microRNA-target interactions. *Nucleic Acids Res*;53(D1):D147–d156.

Ding, Y.*, et al.* (2022) MLRDFM: a multi-view Laplacian regularized DeepFM model for predicting miRNA-disease associations. *Briefings in Bioinformatics*;23(3).

Li, J.*, et al.* (2021) SVDNVLDA: predicting lncRNA-disease associations by Singular Value Decomposition and node2vec. *BMC Bioinformatics*;22(1):538.

Liu, M.*, et al.* (2019) Circbank: a comprehensive database for circRNA with standard nomenclature. *RNA Biol*;16(7):899–905.

Pan, S.*, et al.* Adversarially regularized graph autoencoder for graph embedding. In, *Proceedings of the 27th International Joint Conference on Artificial Intelligence*. Stockholm, Sweden: AAAI Press; 2018. p. 2609–2615.

Paszke, A.*, et al.* PyTorch: an imperative style, high-performance deep learning library. In, *Proceedings of the 33rd International Conference on Neural Information Processing Systems*. Curran Associates Inc.; 2019. p. Article 721.

Saito, T. and Rehmsmeier, M. (2015) The precision-recall plot is more informative than the ROC plot when evaluating binary classifiers on imbalanced datasets. *PLoS One*;10(3):e0118432.

Sheng, N.*, et al.* (2022) Multi-channel graph attention autoencoders for disease-related lncRNAs prediction. *Briefings in Bioinformatics*;23(2).

Sheng, N.*, et al.* (2023) Multi-task prediction-based graph contrastive learning for inferring the relationship among lncRNAs, miRNAs and diseases. *Brief Bioinform*;24(5).

Shi, Z.*, et al.* (2021) A representation learning model based on variational inference and graph autoencoder for predicting lncRNA-disease associations. *BMC Bioinformatics*;22(1):136.

Subramanian, A.*, et al.* (2005) Gene set enrichment analysis: a knowledge-based approach for interpreting genome-wide expression profiles. *Proc Natl Acad Sci U S A*;102(43):15545–15550.

Wang, P.*, et al.* (2022) LncACTdb 3.0: an updated database of experimentally supported ceRNA interactions and personalized networks contributing to precision medicine. *Nucleic Acids Res*;50(D1):D183–d189.

Wu, Q.-W.*, et al.* (2021) GAERF: predicting lncRNA-disease associations by graph auto-encoder and random forest. *Briefings in Bioinformatics*;22(5).

Yao, D.*, et al.* (2018) Circ2Disease: a manually curated database of experimentally validated circRNAs in human disease. *Sci Rep*;8(1):11018.

Zhang, X.*, et al.* (2025) Predicting circRNA–disease associations with shared units and multi-channel attention mechanisms. *Bioinformatics*;41(3).

Zheng, Y.*, et al.* (2023) NPInter v5.0: ncRNA interaction database in a new era. *Nucleic Acids Res*;51(D1):D232–d239.
